# INNBC DApp, a decentralized application to permanently store biomedical data on a modern, proof-of-stake (POS), blockchain such as BNB Smart Chain

**DOI:** 10.1186/s12911-024-02498-z

**Published:** 2024-04-25

**Authors:** Jonathan Fior

**Affiliations:** Innovative Bioresearch Ltd, 20-22 Wenlock Road, N1 7GU London, United Kingdom

**Keywords:** DApp, Web3, DeSci, DeFi, Crypto, Token, Blockchain, INNBC

## Abstract

**Background:**

A blockchain can be described as a distributed ledger database where, under a consensus mechanism, data are permanently stored in records, called blocks, linked together with cryptography. Each block contains a cryptographic hash function of the previous block, a timestamp, and transaction data, which are permanently stored in thousands of nodes and never altered. This provides a potential real-world application for generating a permanent, decentralized record of scientific data, taking advantage of blockchain features such as timestamping and immutability.

**Implementation:**

Here, we propose INNBC DApp, a Web3 decentralized application providing a simple front-end user interface connected with a smart contract for recording scientific data on a modern, proof-of-stake (POS) blockchain such as BNB Smart Chain. Unlike previously proposed blockchain tools that only store a hash of the data on-chain, here the data are stored fully on-chain within the transaction itself as “transaction input data”, with a true decentralized storage solution. In addition to plain text, the DApp can record various types of files, such as documents, images, audio, and video, by using Base64 encoding. In this study, we describe how to use the DApp and perform real-world transactions storing different kinds of data from previously published research articles, describing the advantages and limitations of using such a technology, analyzing the cost in terms of transaction fees, and discussing possible use cases.

**Results:**

We have been able to store several different types of data on the BNB Smart Chain: raw text, documents, images, audio, and video. Notably, we stored several complete research articles at a reasonable cost. We found a limit of 95KB for each single file upload. Considering that Base64 encoding increases file size by approximately 33%, this provides us with a theoretical limit of 126KB. We successfully overcome this limitation by splitting larger files into smaller chunks and uploading them as multi-volume archives. Additionally, we propose AES encryption to protect sensitive data. Accordingly, we show that it is possible to include enough data to be useful for storing and sharing scientific documents and images on the blockchain at a reasonable cost for the users.

**Conclusion:**

INNBC DApp represents a real use case for blockchain technology in decentralizing biomedical data storage and sharing, providing us with features such as immutability, timestamp, and identity that can be used to ensure permanent availability of the data and to provide proof-of-existence as well as to protect authorship, a freely available decentralized science (DeSci) tool aiming to help bring mass adoption of blockchain technology among the scientific community.

## Introduction

### Blockchain network and its main features

**Fig. 1:**
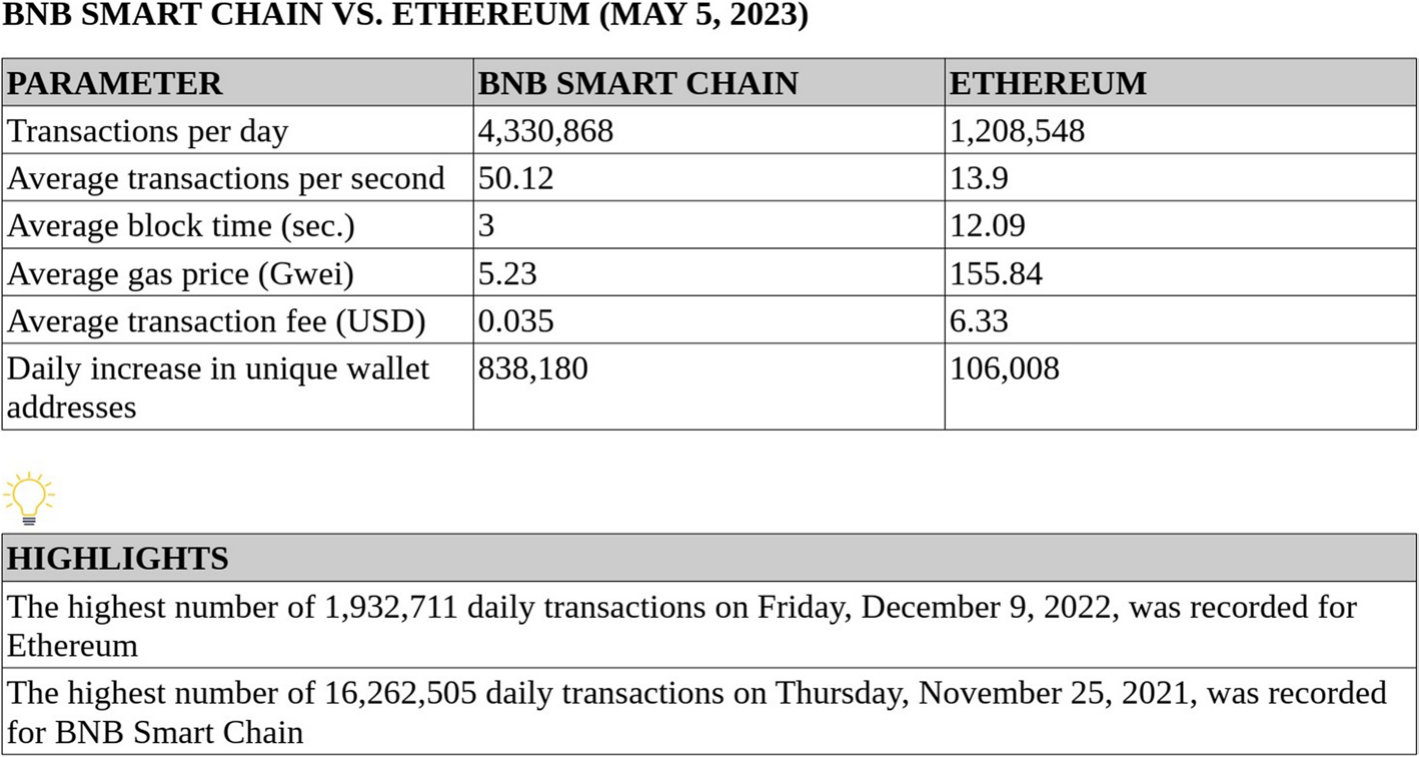
Daily network activity of Ethereum blockchain vs. BNB Smart Chain blockchain recorded on May 5, 2023. Data were sourced from the respective chain explorers (https://etherscan.io/ and https://bscscan.com/). Average transaction fee is intended as a standard transaction for the transfer of the native chain currency (ETH/BNB), consuming 21,000 gas units. According to the chain explorer data, the highest number of daily transactions ever recorded for the Ethereum blockchain is 1,932,711 (recorded on December 9, 2022), while that of BNB Smart Chain blockchain is 16,262,505 (recorded on November 25, 2021). This means that the highest average transactions per second (TPS) output recorded for Ethereum is approximately 22TPS, while for BNB Smart Chain it is approximately 188TPS. Accordingly, BNB Smart Chain scored up to 8.5 times better performance than Ethereum in real-world network usage while featuring substantially lower transaction fees

A blockchain is a distributed ledger database, a sequence of data blocks linked together with cryptography, similar to elements of a virtual chain [1–3]. Each time a new block is added to the blockchain, it includes a cryptographic reference to the previous block in the “previous block hash” field of the block header, a timestamp, and transaction data. Each single block in the chain is identified by its own unique hash and points backward to the block before. As such, each “parent” block serves as a foundation for its “child” block, and the sequence of cryptographic hashes linking each child block to its parent block creates a virtual chain that goes all the way back to the first block ever created, the “genesis block” [4, 5]. If we consider a random block in a blockchain, its information cannot be modified without modifying all the previous blocks, making the data impervious to being compromised, especially considering that such information is replicated among thousands of different nodes. This means that once a block has enough generations of blocks following it, we consider such data immutable [6, 7]. This represents one of the most important features of a blockchain: “immutability”. Once some information is posted on a blockchain, it is permanently stored and can never be altered. Another critical feature of a blockchain is the “timestamp”; each time new information is added to a blockchain, updating its state, it provides us with the exact time and date of the event [3]. If we combine the two elements, immutability and timestamp, we can verify that such data were generated at a certain time point and have not been altered since, providing us with a “digital fingerprint” where each block has its own unique and immutable cryptographic hash identifier along with a date of creation. Each change in the state of a blockchain takes place within a transaction. A transaction is an object that describes the transfer of funds between accounts. It is a cryptographically signed data message containing a set of instructions that is initiated by an externally owned account, such as a user wallet, with its own unique private key used to sign the transaction. Transactions are included in blocks and broadcast to the network nodes, which run some validation logic to confirm that the transactions are valid. This requires some time that can range from a few seconds to several minutes or even hours, depending on the different blockchain networks, before we get a notification that the transaction is confirmed. Secondly, after the transaction is confirmed, it returns a “transaction hash”, which uniquely identifies the transaction. In contrast with traditional centralized databases, in which the data are updated instantaneously, in a blockchain we have to wait for the block time to actually see the transaction confirmed and reflect the new value on the network. Blocks are, therefore, containers of transaction data organized as a public ledger, a blockchain. In a “proof-of-work” blockchain, miners are required to solve machine-generated cryptographic problems to have a chance to write a valid block [8]. Only miners can propose new blocks, while nodes can only validate blocks proposed by miners. By contrast, nodes can also function as miners in a “proof-of-stake” blockchain. These nodes are known as “validator nodes”, and in order to take part in the validation process and be rewarded, they must hold and stake a certain amount of the native chain currency [8]. In a blockchain, the majority of nodes must agree to reach a “consensus” in which the network agrees that a block is valid before it can be added to the chain [8]. All nodes are connected to each other and constantly update their data to reflect the latest blockchain state. Because there are usually thousands of different nodes and miners/validators in a blockchain, we define it as a “decentralized” structure. Bitcoin is the oldest blockchain and the first to gain popularity and introduce this technology to a mainstream audience. The Bitcoin genesis block was mined on January 3, 2009, by anonymous developer known under the pseudonym “Satoshi Nakamoto” [9]. It is noteworthy how such information is permanently recorded due to the immutability of a blockchain, and we can verify, at any time, the bitcoin genesis block creation using a chain explorer [10]. One of the greatest limitations of the Bitcoin blockchain is that its use is mostly limited to the processing of BTC transactions, meaning that its main use case is represented by sending, receiving, and storing BTC as a cryptocurrency. The introduction of “smart contracts” with the Ethereum blockchain, launched in July 2015 by computer scientist Vitaly Dmitriyevich “Vitalik” Buterin, was a disruptive innovation in the blockchain space [11]. On the Ethereum blockchain, there are two things that can be posted: transactions and other data. An example of other data would be smart contracts. Conceptually, smart contracts act as virtual contracts running on the blockchain, featuring executable code called “functions”, programmed in the contract as a set of fixed rules to execute agreements between “untrustworthy parties” without the involvement of a trusted third party. They can be defined as accounts controlled by some code instead of being externally owned accounts controlled by humans. This code instructs the smart contract on how to behave. Once deployed on the blockchain, a smart contract has its own unique address, and its code cannot be altered, making it immutable and thereby acting as an unbreakable contract among individuals. A major bottleneck of the Ethereum blockchain has been its inability to scale well, resulting in network congestion and high fees. In addition, Ethereum was launched with a “proof-of-work” (POW) consensus mechanism, which requires high energy consumption for the mining process, and only recently migrated to a modern and energy-efficient “proof-of-stake” (POS) consensus mechanism. This provided the opportunity for other competitors to emerge in the space, offering blockchains with improved scalability with respect to Ethereum. A particularly successful competitor that has recently emerged is the “BNB Smart Chain” blockchain (BSC) [12]. Launched in September 2020 by Changpeng “CZ” Zhao, software engineer and Binance company founder, BNB Smart Chain (formerly known as Binance Smart Chain) is designed to be fully compatible with the Ethereum Virtual Machine (EVM), featuring a modern proof-of-stake (POS) consensus mechanism along with faster and cheaper transactions. This resulted in a daily transaction volume for BNB Smart Chain that surpassed that of Ethereum (Fig. 1). Currently, a major application of blockchain technology and smart contracts is in finance, with the issuing of cryptocurrencies as well as the introduction of “DeFi” (decentralized finance) [13], blockchain applications that feature complex financial use cases. High-profile figures in the blockchain industry, such as Anndy Lian [14], intergovernmental blockchain adviser, believe that DeFi has a role in the future financial markets, especially because with DeFi, “anybody can apply for a loan”, with no need for a bank or credit checks, just a digital wallet and a crypto asset as collateral [15]. Although the major use case for blockchain technology is currently in finance, we strongly believe that such technology can bring disruptive innovation to the biomedical research field as well, and its potential applications and use cases in science have been largely underestimated.

## The potential of blockchain technology to permanently record biomedical data

Several features of a blockchain, such as immutability, decentralization, security, timestamping, and identity, can have specific applications for biomedical data. Immutability: biomedical data produced by research studies should be permanently stored and never altered. In a blockchain, all data are replicated among thousands of nodes, never ceasing to exist and always staying on the blockchain. This also makes a blockchain censorship-resistant, meaning that information cannot be obscured once posted on-chain, making it an ideal application for biomedical data. Decentralization: a blockchain is designed to be distributed and synchronized across a public network made up of thousands of nodes, making data freely accessible to anyone and not controlled or dictated by a single centralized entity. We believe that scientific data should be publicly shared and not hidden behind a firewall. Security: the types of transactions that can be performed are strictly defined in advance by “smart contracts”, preventing fraudulent data from being added to the chain and ensuring the integrity of the database, as it would require an incredibly large amount of computational power to compromise thousands of nodes. By contrast, it would be much easier to compromise a traditional centralized database running on a centralized server, indicating that decentralizing the data can add an additional layer of protection, ensuring data integrity. Timestamping and identity can provide us with undeniable proof of authorship. Normally, it can be challenging to produce such evidence, particularly in cases where work is carried out in large teams, which can result in disagreements over authorship. On the other hand, if the data, including authorship information, are recorded on a blockchain, the timestamp can establish that the data were created on a certain day and time, giving each author a clear and unambiguous reference. Specifically, recording data on a blockchain provides us with a temporal and permanent track of their creation, allowing us to identify subsequent falsifications and revisions. Furthermore, a blockchain can provide us with the identification and authentication of the author and his or her institution. For instance, each author can be associated with a unique personal wallet so that only the transactions generated by that specific wallet are considered legitimate transactions produced by the author and his or her institution. The author can link his or her official blockchain wallet address to his or her verified social media accounts, official website, and official institution page. As a result, it will be impossible for a malicious person to impersonate the author on the blockchain because the author's off-chain information will validate his or her on-chain identity. Another consideration is how we can verify that the party posting data on-chain provides us with valid data. For instance, let us assume that a company selling laboratory reagents is recording on-chain every single transaction and shipment of its products to its clients. If we want to verify that the on-chain data are real, we can reach out to the clients and ask for confirmation that these transactions are indeed real. A blockchain is, in fact, a timeline of data storage that provides us with a complete temporal record of events that we can cross-check with off-chain information. By cross-checking the on-chain data with the off-chain information, we can determine if the party posting data on-chain is providing valid data. Given that all data on a blockchain are public and each event is accurately time-stamped, posting all the information on a blockchain would make it more difficult for a party to hide fraudulent activity. Here, we assumed that blockchain technology has a valuable application in biomedical research for generating an immutable, decentralized record of biomedical data that can be useful for the aforementioned use cases and developed a decentralized application (DApp) for making such features easily accessible to the scientific community.

## Current underutilization of blockchain technology in biomedical research and healthcare

Analyzing the current literature on the use of blockchain technology in healthcare and biomedical research highlights a scenario in which this technology is largely underutilized, especially in terms of decentralizing the storage and sharing of data. In fact, in most blockchain tools proposed in the scientific literature, the actual data are stored off-chain, with only a hash of the data stored on a blockchain [16–18]. Most likely, they start from the consideration that their data may be too large to fit on a blockchain due to its nature of replicating data among thousands of nodes. They use what is defined as a “blockchain anchor” instead, a unique digital fingerprint of given data generated by hashing the original data with a consistent hashing function such as SHA256. This representation of the data is stored on-chain, while the original data are stored off-chain. To verify their integrity, the off-chain data are compared against the corresponding hash value stored on-chain. This seems to be the most common application of blockchain technology for healthcare data [16–18]. However, this approach does not feature decentralized storage for the main data, meaning that if the off-chain documents are lost, they are lost forever. This approach can be used for proof-of-validity and proof-of-existence, but it cannot ensure the permanent availability of the raw data. By contrast, we propose the strategy of storing the raw data on-chain on a permissionless public network such as BNB Smart Chain, presenting a truly decentralized storage solution that takes full advantage of blockchain technology. Such a strategy has the additional benefit of avoiding the step of using a blockchain anchor and having to verify every single document with its hash because the authenticated data are already fully stored on-chain. This can save a considerable amount of time and complexity. Another sign of the current underutilization of blockchain tools in biomedical research and healthcare is the lack of functioning decentralized applications in the literature, most of which presents conceptual studies or tutorials with unfinished code, lacking an actual functioning tool [19]. By contrast, we provide a finished and fully functional DApp directly linked in the present study.

## INNBC DApp: a Web3 and DeSci tool providing true decentralized storage for biomedical data

**Fig. 2:**
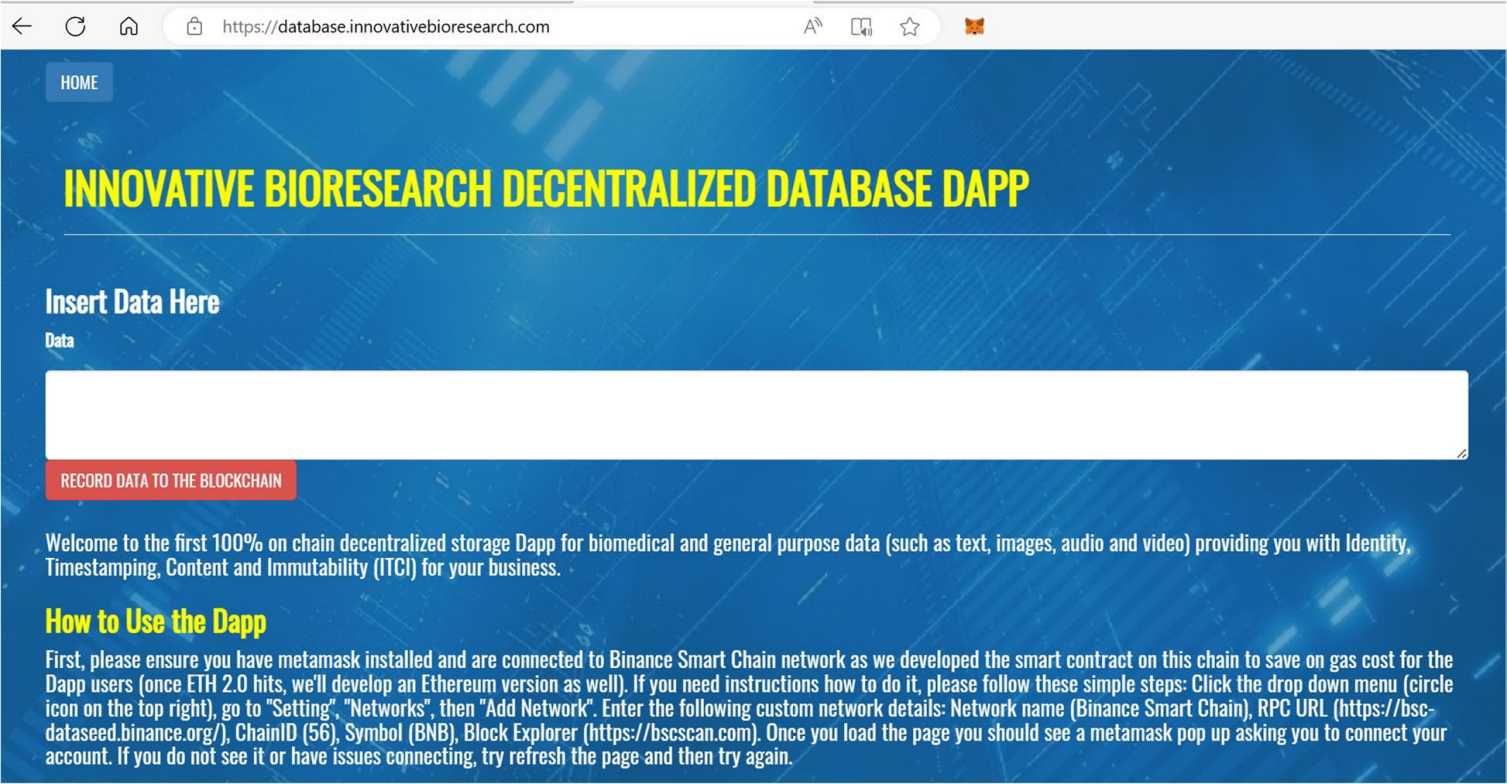
INNBC DApp user interface (UI). We developed a clean and simple UI where users just need to paste the data as text in the input field and press a button to generate a transaction that records the data on the BNB Smart Chain blockchain

“Web3”, also known as “decentralized web”, is a new platform for producing applications that take advantage of blockchain technology and expand their functions beyond what is normally possible with traditional centralized software solutions. “DeSci” stands for “decentralized science”, a term used to define crypto projects, such as INNBC, that aim to introduce real-world use of Web3 in scientific research [20]. Currently, INNBC (InnovativeBioresearchCoin) is an ERC20 token running on the Ethereum blockchain, issued in May 2018, representing the tokenization of our biomedical research and expanding its development [21]. We strongly believe in the potential of blockchain technology to disrupt pharma by decentralizing many aspects of biomedical research, from the funding of the research to the storage and sharing of data. The first goal of INNBC project was to develop a decentralized application to decentralize the storage and sharing of biomedical data, while the next step will be to develop INNBC Smart Chain, our own blockchain using INNBC as its native currency, which will be devoted to decentralized science [20]. The results of the first project goal are discussed here. Accordingly, the goal of this work is to provide a Web3 application that allows users to easily store, on a public blockchain, scientific text, images, and a variety of other files that may be included in a scientific study. Specifically, we developed “INNBC DApp”, a web-based DApp with very low minimum requirements for increased accessibility that simply requires a web browser with a decentralized Web3 wallet installed (e.g., Metamask). The goal of a decentralized application is to provide a straightforward user interface (UI) for accessing smart contract functions. Therefore, a DApp is essentially a user interface (front-end) running on a local server that enables users to execute the functions of a smart contract (back-end) running remotely on a blockchain. To be more specific, the smart contract contains an “application binary interface” (ABI) code, acting as a layer of translation between the front-end JavaScript code and the back-end “bytecode” of the smart contract. When we designed INNBC DApp, we had several goals in mind. First and most importantly, our aim is to help bring mass adoption of blockchain technology into science by decentralizing the storage and sharing of biomedical data. INNBC DApp features a very simple and straightforward front-end UI, where users just need to paste their data as text in the input field and press a button to store the data on-chain (Fig. 2). Another critical feature of INNBC DApp is true decentralized storage, which means that the data are actually stored fully on-chain to achieve true decentralization and immutability. In this regard, previous literature on the use of blockchain technology in healthcare mainly proposes the use of smart contracts for storing on-chain just a hash of the data while storing the actual data off-chain [16–18]. By contrast, we store all data on-chain, as we developed a specific smart contract that can generate a transaction and store the desired data as “transaction input data”, which are additional data that can be attached to a transaction. Specifically, a transaction has an “input data field” that can include two types of data: function call data and arbitrary data. Function call data are used to call a specific smart contract function, and they include the function signature for the parameters required by the function. Arbitrary data are not executed by the Ethereum Virtual Machine (EVM) and translate into human-readable text in UTF-8 format. Arbitrary data are where we include the data that we post on the blockchain with INNBC DApp. As such, our design choice was to store the data not directly in the smart contract but within the transaction itself. We believe this is a clever way of storing biomedical data on a blockchain. Another important aspect we had in mind was versatility. Biomedical data can include not only plain text and numbers but also images and, to a lesser extent, audio and video. Therefore, a solution for storing different files on-chain is needed. We achieve this goal by encoding files with Base64, a binary-to-text encoding algorithm, and then uploading them as a string of text using INNBC DApp. In the following sections, we assess the feasibility of storing text and images from scientific publications on a blockchain, analyzing costs and limitations. Any time users want to invoke a function that is intended to change some data on the blockchain, they are going to invoke it by sending a transaction to the contract instance that targets the specific function, which involves paying a transaction fee. In fact, every time a transaction is sent to the network, there is a gas price attached to it. The gas system is meant to measure how much work is executed by the code in terms of how much it costs to run that code. Because our goal is to allow users to store data with low fees, we have chosen BNB Smart Chain for the initial release of our DApp while also planning to release an Ethereum version once the chain is fully upgraded to ETH 2.0 with the introduction of sharding [8] to lower the transaction fees. BNB Smart Chain (BSC) is an ideal blockchain for developing decentralized applications because it is essentially a hard fork of the Go Ethereum (Geth) protocol. As such, all the smart contracts and DApps built on BSC are compatible with the Ethereum Virtual Machine (EVM), while taking advantage of POS and handling up to 8.5 times more transactions per second, resulting in significantly lower transaction fees than Ethereum (Fig. 1).

## Materials and methods

### Coding language

Using a combination of HTML, JavaScript, and Solidity programming languages, we developed a web-based DApp featuring a front-end UI (available at https://database.innovativebioresearch.com/) connected with a smart contract that we deployed on the BNB Smart Chain (0x7f92320f7f9f20485381f9e7dc3459bc776e2728). We used the Web3.js library, an Ethereum JavaScript API built by the Ethereum Foundation that talks to the blockchain using the “JavaScript Object Notation-Remote Procedure Call” (JSON-RPC) protocol, to connect the front end to the smart contract and call its functions. The smart contract source code is publicly available on the contract page on the BSC chain explorer (https://bscscan.com/address/0x7f92320f7f9f20485381f9e7dc3459bc776e2728).

### How to use INNBC DApp

**Fig. 3:**
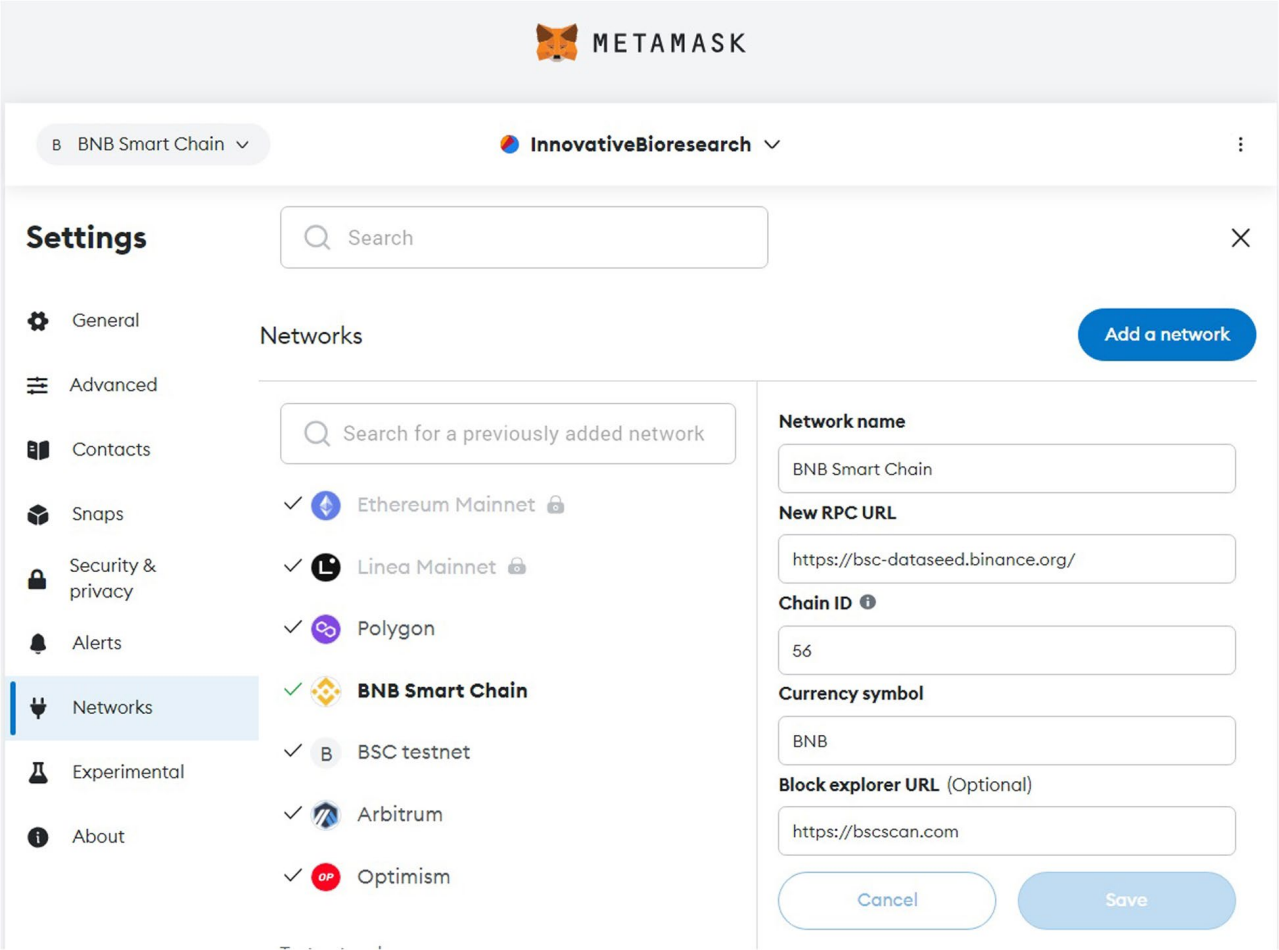
Parameters for configuring the Metamask browser extension (https://metamask.io/) for the BNB Smart Chain blockchain network in order to interact with INNBC DApp

**Fig. 4:**
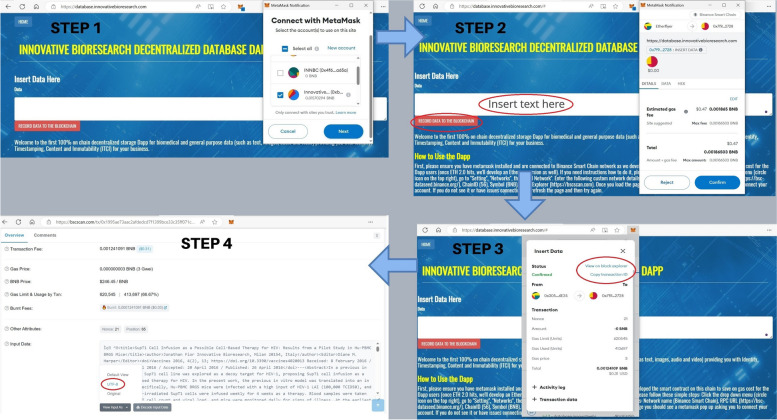
Step-by-step illustration of how to use INNBC DApp for storing data on-chain. Step 1: go to the DApp page (https://database.innovativebioresearch.com/) and connect your Metamask wallet to the DApp. After the wallet is connected, please refresh the page to ensure that all components are loaded. Step 2: Paste the text you want to record in the input field, press the “RECORD DATA TO THE BLOCKCHAIN” button, and sign the transaction when the Metamask pop-up appears asking to confirm it (if nothing happens, refresh the page and try again). Step 3: browse the transaction history on Metamask and find your transaction; from there, you can copy the transaction ID (“Copy transaction ID”) and save it as your permanent link to the data on the blockchain or check the transaction on the chain explorer (“View on block explorer”). Step 4: Click on “View on block explorer”; from the transaction page, click on “Click to see More”, “View input as”, select “UTF-8”, and in the “Input Data” field, you can find your data

In order to successfully use the DApp, a web browser (such as Firefox, Chrome, or Edge) with a Web3 wallet extension such as Metamask (https://metamask.io/) installed is required, along with proper configuration of Metamask for the BSC blockchain. To configure Metamask for BSC blockchain, click the drop-down menu (three vertical dots on the top right), go to “Settings”, “Networks” and “Add Network”, then enter the following custom network details: Network Name (BNB Smart Chain); RPC URL (https://bsc-dataseed.binance.org/); Chain ID (56); Symbol (BNB); Block Explorer (https://bscscan.com). You can now select the newly created “BNB Smart Chain” as your network on the top left menu (Fig. 3). Once the DApp page is loaded (https://database.innovativebioresearch.com/), you should see a Metamask pop up asking to connect your account. After you have successfully connected your wallet, refresh the page to ensure that all components are loaded. Now, notice a field for inserting the data and a button labeled “record data to the blockchain”; simply paste the desired text in the input field, then press the button and approve the transaction (if nothing happens, refresh the page and try again to ensure all components are loaded). You can find the TxID (transaction ID) of your transaction in the Metamask transaction history, representing your unique reference to the data on the blockchain. The data can be retrieved from the transaction page on the chain explorer, which is also linked in the Metamask transaction history or reachable by searching the TxID on the chain explorer (https://bscscan.com/). Once you are on the explorer page, click on “Click to see More”, “View input as”, select “UTF-8”, and in the “Input Data” field, it is finally possible to retrieve the data. All these steps are illustrated in Fig. 4. Even in the remote scenario in which the DApp page would go offline, it is still possible to reach the smart contract, which “lives forever” on the blockchain, from the contract page (https://bscscan.com/address/0x7f92320f7f9f20485381f9e7dc3459bc776e2728) and call its functions. In addition to the contract deployed on the BSC “mainnet”, the actual blockchain, we also deployed a test contract on the BSC “testnet” (https://testnet.bscscan.com/address/0x5010b4afe9f3a25cc45522c339bce2f78cfb0798). The testnet is a testing “sandbox” environment for developers used strictly for testing purposes, where currencies do not hold any actual value and one can source free “test BNB” (from a faucet such as https://testnet.bnbchain.org/faucet-smart) for testing smart contracts and estimating the cost and outcome of any operation before paying actual fees. In this study, fees are expressed as BNB, and when indicated using the “$” sign, they represent the corresponding U.S. dollar value based on the BNB/USD exchange ratio at the time of transaction.

## Results

### Storing on-chain a previously published research study as raw text

As a possible attempt to store scientific data on-chain, we decided to store one of our previously published research studies [22] by directly uploading the data as raw text. This means that instead of uploading the data as files, we directly uploaded the raw article text into the transaction input data. The study involved infecting a humanized mouse model with HIV to test whether the infusion of irradiated SupT1 cells could act as a decoy target for the HIV virus to prevent infection of primary CD4+ T cells. Text and figures constitute the article's two primary components. In this instance, the figures are essentially high-resolution graphs providing a visual representation of numerical data. We assumed that storing the raw numerical data in a single transaction instead of the actual figure files would save in terms of gas fees and explored such a strategy. In this regard, considering that authors will also handle their unpublished data, it might be a good idea to record the raw numerical data on a blockchain in order to create an undeniable proof of authorship before sharing the corresponding figure files publicly. This will help protect their work from being stolen or altered. We decided to store the figure data in a first transaction and the text of the research article in a second transaction, in which we also referenced the TxID of the figure data. In order to store text and numbers in a readable way, we decided to use tags similar to HTML to describe the data (e.g., <ABSTRACT >insert text here</ABSTRACT>, <FIGURE DATA >insert data here</FIGURE DATA>). Following the steps illustrated in Fig. 4, we posted on-chain the raw numerical data of the figure files, generating a transaction (https://bscscan.com/tx/0x217268b23829f4ae1a84b258826578658a863fa5abc75df7a57315379fd6b09c), from which we can also copy the transaction hash (0x217268b23829f4ae1a84b258826578658a863fa5abc75df7a57315379fd6b09c), which provides us with a permanent reference to the data on the blockchain. This resulted in a transaction cost of 0.00067BNB/0.17USD for storing the raw numerical data. Next, we uploaded the article text on a successive transaction (https://bscscan.com/tx/0x1995ae73aac2afdedcd7f1399bce33c35ff071cb62786d1c84d0dce012e0d564), in which, as previously mentioned, we also referenced the TxID of the figure data. Storing the article text resulted in a fee of 0.0012BNB/0.31USD, with a total cost of approximately $0.48 for storing the article text and figure data. We believe that it is a negligible cost to permanently store the data on a blockchain, which provides us with immutability, timestamping, proof of authorship, and identity. For reference, the article text has a word count of 3,714, while the figure data have a word count of 2,237. As previously mentioned, this can be especially useful for preprints and unpublished data but also for storing additional content linked to the main paper in peer-reviewed articles (such as experiment raw numerical data, which are what we used here for storing the figure data). Although it could be argued that storing them on preprint services is free, they do not offer the decentralized features of a blockchain.

### Storing actual images on-chain

**Fig. 5:**
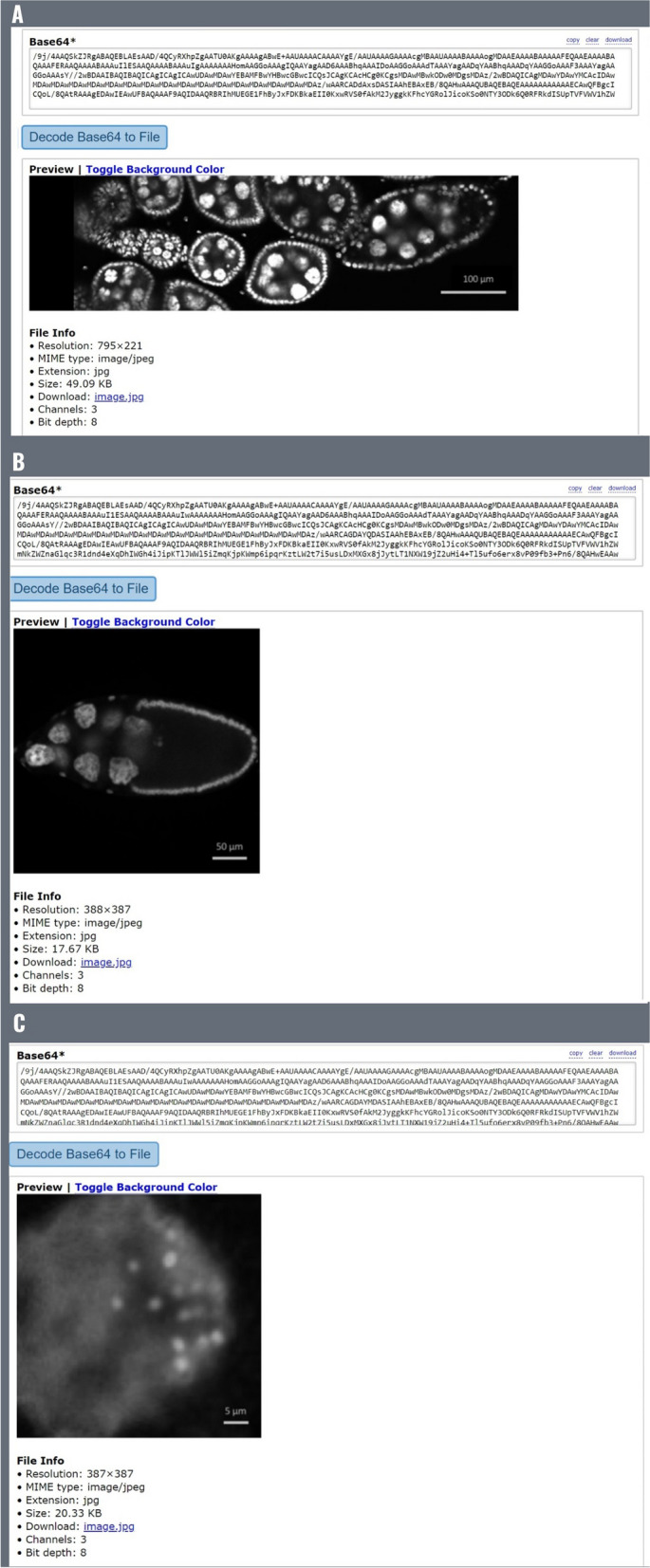
Retrieving the original image files from Base64-encoded data. Here, we show how we reconstructed the three original images (**A**, **B**, **C**) from the on-chain data using a free Base64 online tool (https://base64.guru/)

**Fig. 6:**
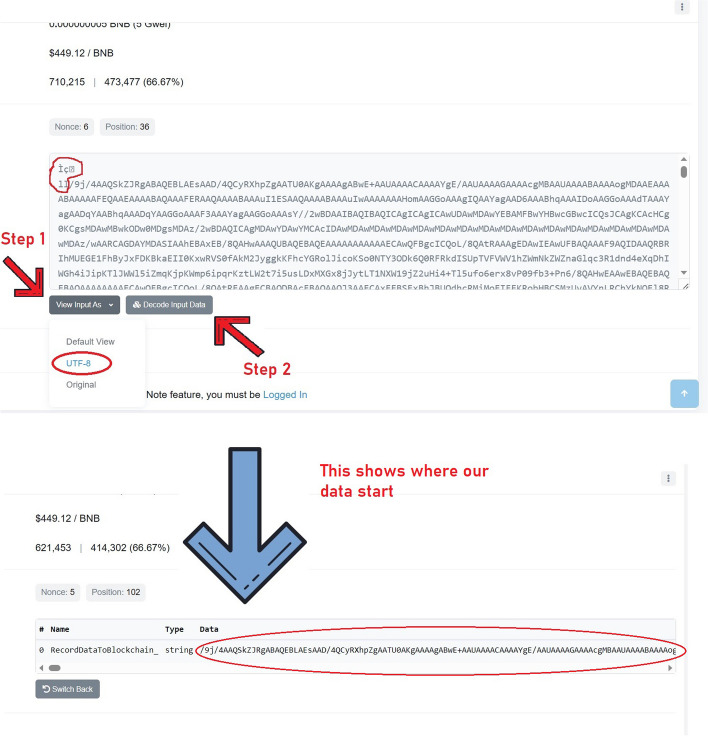
When retrieving the data from the chain explorer page by pressing “View Input as UTF-8”, we need to remove some extra characters at the beginning of the text. To recognize them, pressing “Decode Input Data” will show us exactly where our data start (figure bottom), and we can easily exclude these extra characters when copying the text

**Fig. 7:**
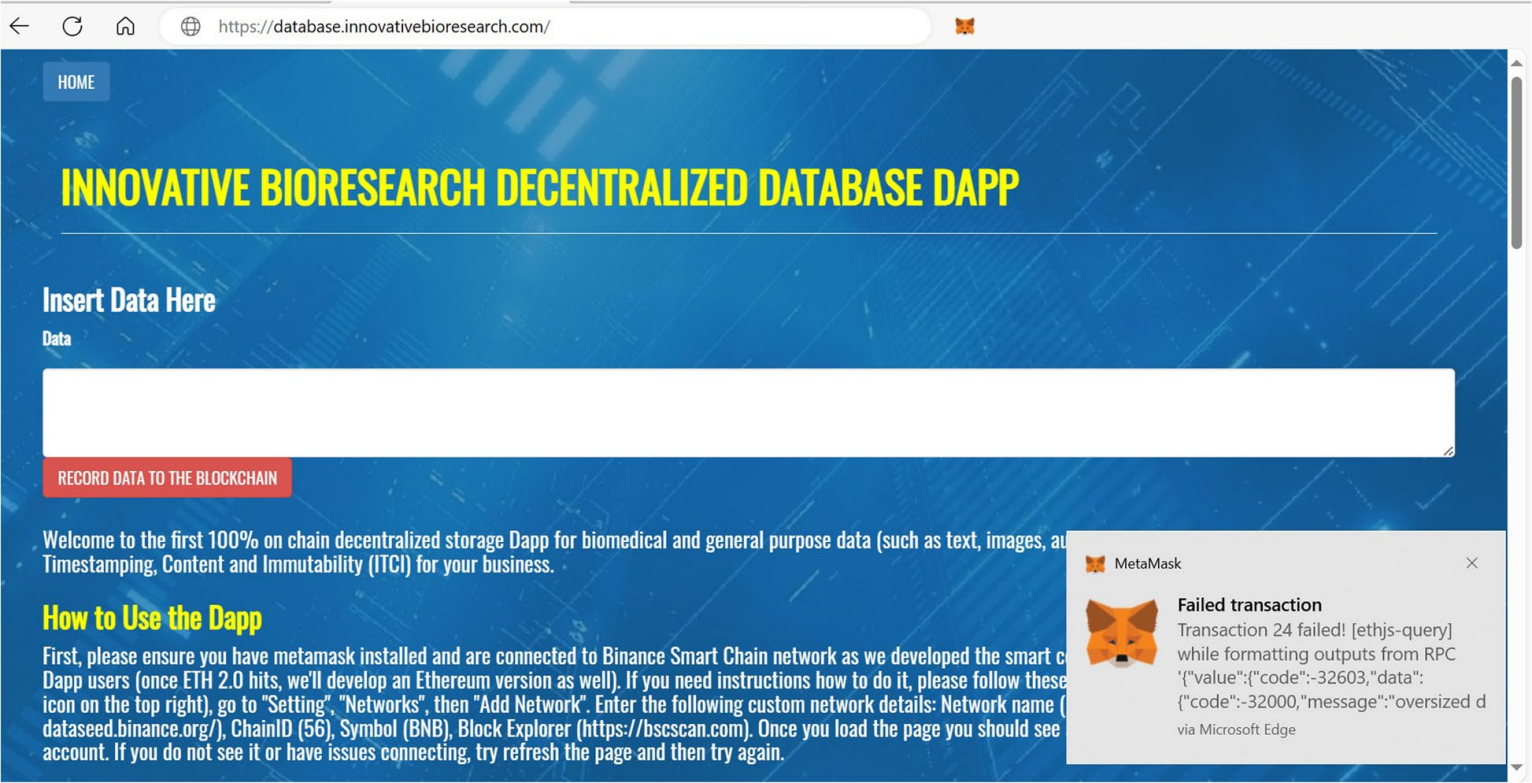
Oversized data error. This error was generated when the transaction did not go through due to the data exceeding 95KB in size (we tested 1KB increments and started getting this error from a 96KB file size). Considering that there is an increase in size of 33% with respect to the original data due to the Base64 encoding, the actual size limit would be approximately 126KB for each single upload

**Fig. 8:**
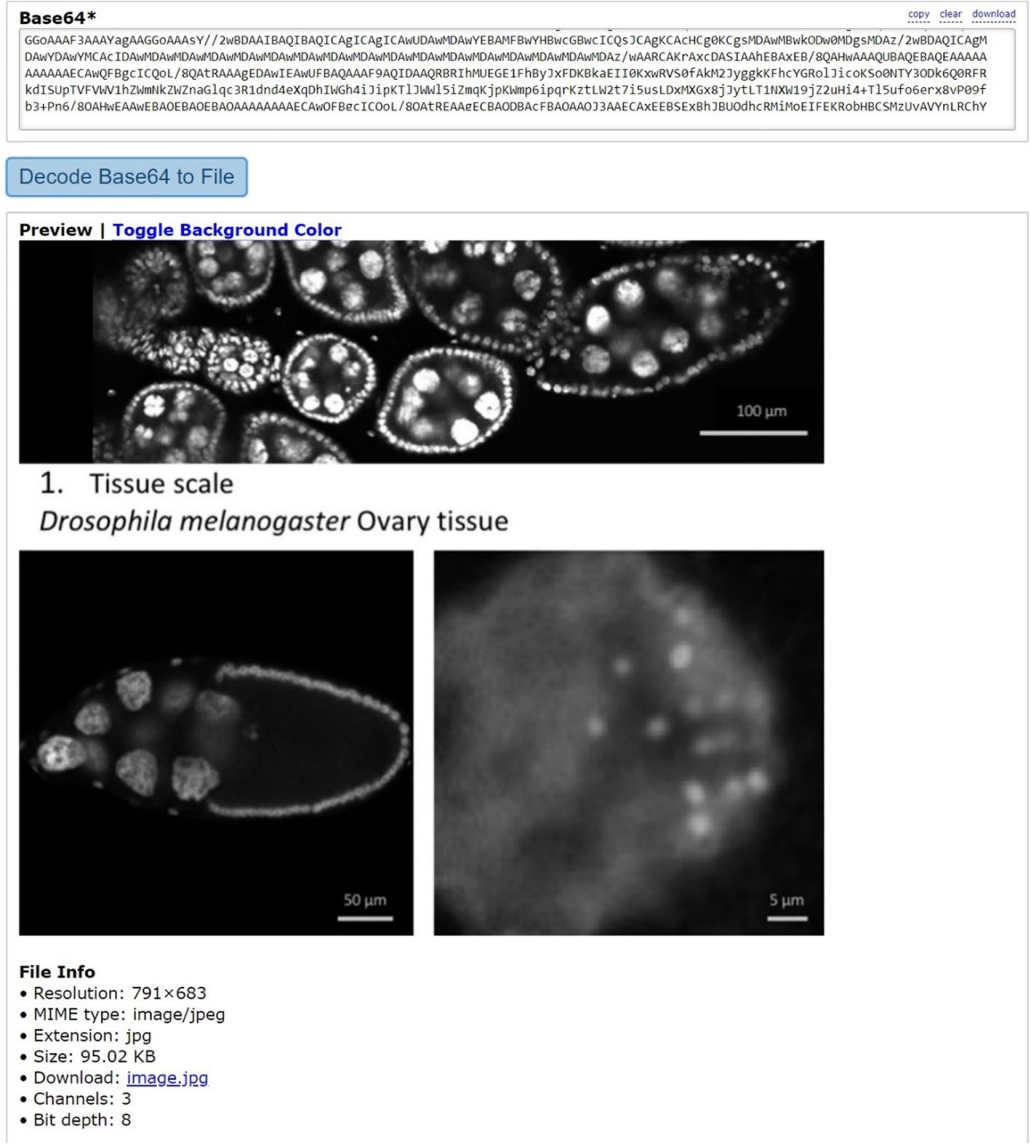
Reconstructing the original image file from Base64-encoded data using a free Base64 online tool (https://base64.guru/). Here we reconstructed the image file for the 95KB image, which according to our tests was the maximum allowed amount of original data encoded to Base64 for a single transaction. As previously mentioned, this image is sourced from a previously published work (doi: 10.1371/journal.pbio.3001161.g003)

Previously, we stored the article figure data not as figure files but as their raw numerical data, given that they are line graphs summarizing such data, and we included all of them in a single transaction to save on transaction fees. However, this is not possible with figures containing actual photographs (e.g., gel images, microscope images, diagnostic images, radiology images, fluorescence images), for which we need to store the actual image file. We therefore decided to assess whether it is possible to store actual image files on-chain. Using a binary-to-text encoding algorithm such as Base64, it is possible to convert a file into a stream of 64 ASCII characters, which can then be stored on-chain using our DApp. With this procedure, we attempted to store images sourced from a paper discussing how to properly create figures suitable for scientific publications in order to be representative of generic scientific images [23]. Specifically, we used the paper’s figure 3A (10.1371/journal.pbio.3001161.g003). Part A of this figure illustrates Drosophila melanogaster tissue at three different microscopic scales, from which we extracted three singular images, resulting in three files weighing 49KB, 17.67KB, and 20.33KB, and stored them on-chain, generating three different transactions: https://bscscan.com/tx/0xdd4d10e73572200759a9ced8e8ef5f6d401092af8f67475c39fcf9d2f870360d (0.005BNB/2.51USD fee); https://bscscan.com/tx/0x4b651846b43f7424010a569f47ba7812318c21c3fa98b65d30d28cce4307184d (0.002BNB/0.93USD fee); https://bscscan.com/tx/0x37fc74bbc631109f2a114721687ce31f2771ac30b2b7e1c390da4a2981222a09 (0.0023BNB/1.06USD fee). We then converted the data back from Base64 to the original file format, reconstructing the three original image files (Fig. 5). There are a few external characters that need to be deleted at the beginning of the text during this procedure, but they are easily recognizable by pressing “Decode Input Data”, which will show us exactly where our data start, making it simple to delete them (Fig. 6). This demonstrates that scientific image storage on a blockchain is possible. At this point, we made some scalability tests, progressively increasing the file size, to find the limitations. We discovered that we could upload images up to 95KB in size, whereas starting from 96KB, we would get error “oversized data”, meaning that we are attempting to store more data than the chain allows in a single transaction (Fig. 7). However, it is important to consider that Base64 encoding results in a 33% file increase, meaning that the actual theoretical limit would be around 126KB (95KB plus 33%). Accordingly, we successfully uploaded on-chain a 95KB image as the largest possible size allowed by INNBC DApp (https://bscscan.com/tx/0x6831da5deb68315e37d238699c960a95f16623250c0a49c3e61f0c4768bec4a7). This 95KB image was generated by cutting the whole part A of the same original figure used previously (10.1371/journal.pbio.3001161.g003) [23]. As expected, we also successfully reconstructed the original image file from the on-chain data (Fig. 8). To overcome this limitation and upload images larger than 95KB, a possible workaround is to split the data into smaller chunks, meaning that it is theoretically possible to upload images of any size. Considering that the cost for storing 95KB was 0.01BNB/2.66USD, storing 1 megabyte of image data should cost approximately 0.1BNB/26USD, a reasonable cost for permanently storing data on a public blockchain. However, due to BNB and gas price fluctuations, the cost of storage can vary depending on the time of transaction, and we later experienced even lower fee costs, as low as 0.06BNB/14USD per MB.

### Storing audio and video data on-chain

**Fig. 9:**
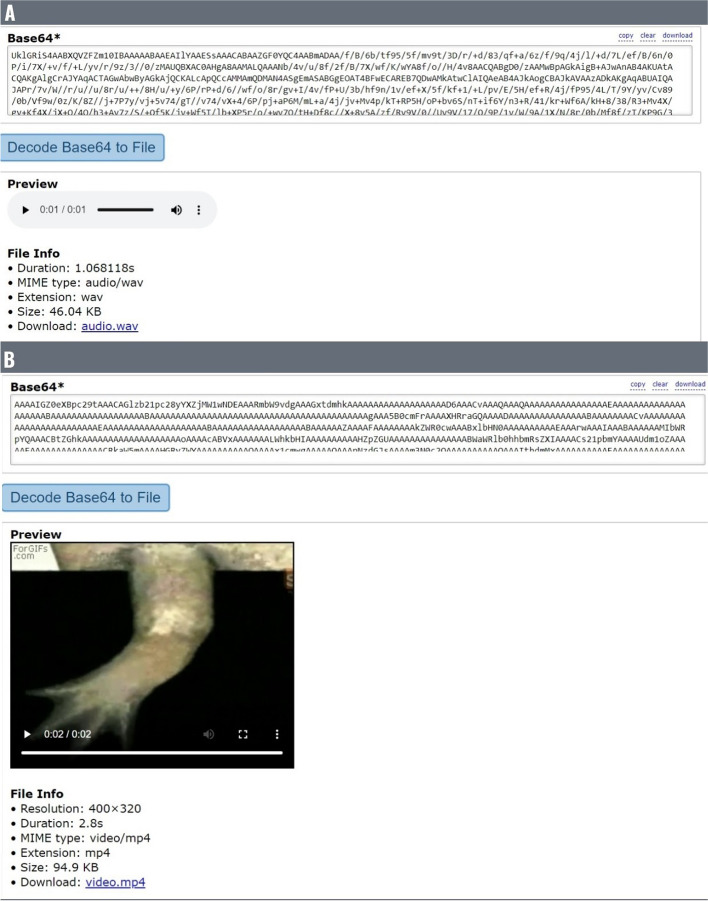
Audio and video files on the blockchain. Here, we stored on-chain a 46KB wav audio file (**A**) and a 94.9KB mp4 video file (**B**), which were encoded to Base64 and then decoded back to file using a free Base64 converter tool (https://base64.guru/), with which we can potentially store on-chain a very large number of different file formats with INNBC DApp

We also wanted to assess the possibility of storing additional file formats such as audio and video on the Binance blockchain using INNBC DApp, given that these types of data might also be included in research studies. Keeping the previous limitations in mind, we stored files less than 95KB in size. We stored on-chain a 46KB wav audio file featuring a sound effect at a cost of 0.005BNB/2.62USD (https://bscscan.com/tx/0x7824740920767e8265f2a571004b5f569cc45bea49f27d526a9cdfc67415b126), and a 94.9KB mp4 video file showing salamander limb regeneration, a phenomenon that may hold the key to developing novel regenerative and anticancer therapies [24], at a cost of 0.01BNB/5.37USD (https://bscscan.com/tx/0x0070b6e0a7c73da987fdc0449bbbc3e6278a41972fe2f3ea79e3b216d34258e0). We successfully reconstructed the original files from the Base64 data stored on-chain (Fig. 9). This demonstrates that the present procedure also works for these file formats. To the best of our knowledge, this is the first attempt to store audio and video data on the BNB Smart Chain blockchain. Notably, it should be possible to convert a very large number of additional file formats to Base64 and store them on-chain.

### Storing whole scientific articles on the blockchain

In order to provide a more thorough analysis of the costs associated with using the proposed tool for the intended use case, after demonstrating that it is possible to use INNBC DApp to store on-chain different types of files we may find in a scientific article, we decided to store several complete research articles [25–31]. Starting with the first paper on the list [25], a randomly chosen article from the “BMC Medical Informatics and Decision Making” journal, the article comprises two fundamental elements, text and figures, that were posted on the blockchain separately. Previously, we directly stored the raw article text, losing the original formatting, and “described” it with tags similar to HTML to label the different sections and subsections to preserve the information. Editing all the text and separating it into tags can be time-intensive. To make this process easier and faster, we decided to upload the text as a document file instead of directly as text. This approach has the advantage of being much faster and requiring minimal effort for preparing the files for uploading; however, it also involves the additional step of converting the file to Base64 and then decoding it back to file instead of having it directly as readable text in the transaction data. We compressed the article images in WebP format, which represents an optimal format for reducing size and preserving image quality, and stored the text as a Word DocX file. Accordingly, we produced the following files that were posted on the blockchain: article's figure 1 (size: 21.9KB, TxID: https://bscscan.com/tx/0xd060462c327a9bd73c669ef964958cecee0a1c221655d487968689ab2a89910b, cost: 0.0015BNB/0.38USD), article's  figure 2 (size: 28.2KB, TxID: https://bscscan.com/tx/0x8ed2fa830dffade1a49080bb96193fdba2c4c184d093fde1297f87ac731cdefb, cost: 0.0019BNB/0.48USD), article's figure 3 (size: 44.3KB, TxID: https://bscscan.com/tx/0x33421f33aaebdf65a0d4138f6fcb98c8d5b5d61eb7720b1173ba18beaa5b76be, cost: 0.003BNB/0.75USD), article's figure 4 (size: 42.6KB, TxID: https://bscscan.com/tx/0x1c038801e16cf30f8ccd7254b07caac5d051b57225e836e7119e6bd943f7faa4, cost: 0.0029BNB/0.72USD), article's figure 5 (size: 54.8KB, TxID: https://bscscan.com/tx/0x3f85029d2eace5be808a52f6a04834af179fb6d52dfd12e30eae3c2dde79380c, cost: 0.0037BNB/0.93USD), and article’s text (size: 41.3KB, TxID: https://bscscan.com/tx/0x034b53b4e3ec62f6d24285871388a4078ce5082b738bf96b7e9eed3bc08bc784, cost: 0.0028BNB/0.58USD). Files were encoded to Base64 with a free online tool (https://base64.guru/) and posted on the blockchain as Base64-encoded text with INNBC DApp. It is important to mention that when decoding Base64, we may need to rename the file with the correct extension. For instance, after decoding the document file, we ended up with a “.bin” file that we renamed to the correct “.docx” extension. The TxIDs of the figure files were included in the article text as previously described. The total cost for storing the whole article data is approximately $3.8, a relatively low cost for ensuring permanent availability of the data on the BNB Smart Chain blockchain. Especially considering that storing data on a blockchain provides us with immutability, timestamping, proof of authorship, and identity. Using WebP format for the image data and storing the article text as a DocX Word document seems to be an excellent combination for on-chain storage, allowing reduced file size, reduced storage cost, and easy preparation of the article files. We suggest that storing data as files may be an optimal solution when dealing with scientific articles to preserve the original formatting and optimize storage cost, while storing articles directly as text may be useful when dealing with simple and short text to be immediately readable on-chain. Using the same procedure, we posted the other articles [26–31] on the blockchain (results reported in Table 1, which summarizes the results of all transactions performed in the study for ease of comparison).

**Table 1:** Storing biomedical data on the BNB Smart Chain blockchain with INNBC DApp. Here is a summary of all data posted on-chain in the study, with a description of the data, the chain explorer link, and the associated gas fee paid for each transaction

Content	Chain Explorer (TxID)	Gas Fee (USD)
**Research article** [22] (https://dx.doi.org/10.3390%2Fvaccines4020013)		
Article text uploaded as raw text (3,714 words)	https://bscscan.com/tx/0x1995ae73aac2afdedcd7f1399bce33c35ff071cb62786d1c84d0dce012e0d564	0.0012BNB ($0.31)
Article figure data uploaded as raw text (2,237 words)	https://bscscan.com/tx/0x217268b23829f4ae1a84b258826578658a863fa5abc75df7a57315379fd6b09c	0.00067BNB ($0.17)
**Research article** [25] **(**10.1186/s12911-023-02200-9**)**		
Article text uploaded as DocX Word document file (41.3KB)	https://bscscan.com/tx/0x034b53b4e3ec62f6d24285871388a4078ce5082b738bf96b7e9eed3bc08bc784	0.0028BNB ($0.58)
Figure 1 uploaded as WebP file (resolution: 893 × 1230; size: 21.9KB)	https://bscscan.com/tx/0xd060462c327a9bd73c669ef964958cecee0a1c221655d487968689ab2a89910b	0.0015BNB ($0.38)
Figure 2 uploaded as WebP file (resolution: 1885 × 1062; size: 28.2KB)	https://bscscan.com/tx/0x8ed2fa830dffade1a49080bb96193fdba2c4c184d093fde1297f87ac731cdefb	0.0019BNB ($0.48)
Figure 3 uploaded as WebP file (resolution: 1944 × 1003 size: 44.3KB)	https://bscscan.com/tx/0x33421f33aaebdf65a0d4138f6fcb98c8d5b5d61eb7720b1173ba18beaa5b76be	0.003BNB ($0.75)
Figure 4 uploaded as WebP file (resoltuion: 1944 × 1042; size: 42.6KB)	https://bscscan.com/tx/0x1c038801e16cf30f8ccd7254b07caac5d051b57225e836e7119e6bd943f7faa4	0.0029BNB ($0.72)
Figure 5 uploaded as WebP file (resolution: 1944 × 1755; size: 54.8KB)	https://bscscan.com/tx/0x3f85029d2eace5be808a52f6a04834af179fb6d52dfd12e30eae3c2dde79380c	0.0037BNB ($0.93)
**Research article** [26] **(**10.1186/s12911-023-02202-7**)**		
Article text uploaded as DocX Word document file (38.4KB)	https://bscscan.com/tx/0x5703a6d497b46307383ae51f90d400f825138b3c5db6cf8ea3514701cfbf3077	0.0026BNB ($0.65)
Figure 1 uploaded as WebP file (resolution: 1000 × 1098; size: 32.6KB)	https://bscscan.com/tx/0xef1ae262e08164bc3e4951dc34259960c1085edb9c3800d612f777e42560c457	0.00224BNB ($0.55)
Figure 2 uploaded as WebP file (resolution: 975 × 1715; size: 26.6KB)	https://bscscan.com/tx/0x693d81a4df7a0dd8aaee74fa949881b46984293373da1ef5e0a6be8af8310568	0.00184BNB ($0.45)
Figure 3 uploaded as WebP file (resolution: 685 × 1204; size: 15.9KB)	https://bscscan.com/tx/0xaecbad1acbd1d1302e3bb6ac09b38909bf60cf3af9f4aa19537f3efe08d120ef	0.00112BNB ($0.28)
Figure 4 uploaded as WebP file (resolution: 975 × 1643; size: 16.2KB)	https://bscscan.com/tx/0xd5b1bf5d300b872841daeb98580bda304d5397d2422e3abf4ea33cdea1423388	0.00114BNB ($0.28)
**Research article** [27] **(**10.1186/s12911-023-02197-1**)**		
Article text uploaded as DocX Word document file (56.9KB)	https://bscscan.com/tx/0x894aab75631022581347d35863269b9e13b1e6082b6d9931096b57ff41f9e67c	0.0038BNB ($0.94)
Figure 1 uploaded as WebP file (resolution: 2008 × 967; size: 52.9KB)	https://bscscan.com/tx/0x12e750ce380da6393f562ec91b9eafc4694f64f5c65efb7ecb9cb51fbcaf2c57	0.0036BNB ($0.87)
Figure 2 uploaded as WebP file (resolution: 975 × 610; size: 16.5KB)	https://bscscan.com/tx/0xcc7066fe48a33cf22356929b4370543a21f673ced7e4fb1b0455eb1b6ea00cad	0.00117BNB ($0.28)
**Research article** [28] **(**10.1186/s12911-021-01498-7**)**		
Article text uploaded as DocX Word document file (48.5KB)	https://bscscan.com/tx/0xac239952cd8240bb8353997619858cd3f2f4a7123200dab30c7cd0e73c40c3d2	0.0033BNB ($0.70)
Figure 1 uploaded as WebP file (resolution: 797 × 1034; size: 46.8KB)	https://bscscan.com/tx/0x375ba5c92202f4ad3befd697daec73429f85a57d85ce7459340dfc09b77d599d	0.0031BNB ($0.78)
Figure 2 uploaded as WebP file (resolution: 800 × 790; size: 42.3KB)	https://bscscan.com/tx/0x49ab8a0b8a56fe95519eb60537095d3e08cb2aefd2ed2eb0b793da79c44cceb3	0.00289BNB ($0.70)
**Research article** [29] **(**10.1172/jci.insight.168110**)**		
Article text uploaded as DocX Word document file (63.2KB)	https://bscscan.com/tx/0x0e745f9272e25f0ddd7d3eb17812a5b20d1bd6b5cb5fc34bc6bf7d017e0d0862	0.0043BNB ($1.04)
Figure 1 uploaded as jpg file (resolution: 1149 × 838; size: 51.7KB)	https://bscscan.com/tx/0xbbdbed81e521aff7f44c1977ec63038389d0b5229a17d572a74a87409ee5dd21	0.0035BNB ($0.86)
Figure 2 uploaded as jpg file (resolution: 1149 × 702; size: 40.4KB)	https://bscscan.com/tx/0x9f0685b57d282d045e3a0f052944c603bafd012db2645e0e288dc490921615ef	0.0027BNB ($0.67)
Figure 3 uploaded as jpg file (resolution: 1149 × 1110; size: 51.2KB)	https://bscscan.com/tx/0x4d821c25d631ea05ae7cc0e1876305118a44074bc6f6e3ae5aec8a52a5263ae2	0.00349BNB ($0.85)
Figure 4 uploaded as jpg file (resolution: 1149 × 1207; size: 58.7KB)	https://bscscan.com/tx/0xf230377c2bbfa2e3e0c48f82ee8aff9eab3a026451519062e71634544ead33f2	0.004BNB ($0.97)
Figure 5 uploaded as jpg file (resolution: 1149 × 623; size: 34.6KB)	https://bscscan.com/tx/0x54ecc5c9c4c7c86287308ef358e878976df8bf4da52adc8c2df9081b483321f5	0.00237BNB ($0.58)
**Research article** [30] **(**10.1007/s13318-020-00640-6**)**		
Article text uploaded as DocX Word document file (56.9KB)	https://bscscan.com/tx/0xa953e4f2de8caa63aa5c64c5c3447877de4e1f18f9f4abae704a623c271f5aff	0.0038BNB ($0.94)
Figure 1 uploaded as WebP file (resolution: 1533 × 1410; size: 75.9KB)	https://bscscan.com/tx/0x4f08a39d4811c224b768c2019de0b650f812cb97e81e3a2877d89847f957db6e	0.00516BNB ($1.25)
Figure 2 uploaded as WebP file (resolution: 1533 × 1393; size: 69.6KB)	https://bscscan.com/tx/0x861639b8103d82d310fd1e2177ac26caff170a5bf622a31e28adf14332f7b67f	0.0047BNB ($1.15)
Figure 3 uploaded as WebP file (resolution: 968 × 724; size: 20.2KB)	https://bscscan.com/tx/0x6a070ac9f4786966e33aa7a2ab3ff0b08fb8d23b3396a2f1c8224bbfb663af63	0.0014BNB ($0.34)
**Research article** [31] **(**10.1186/1472-6947-5-24**)**		
Article text uploaded as DocX Word document file (31.3KB)	https://bscscan.com/tx/0xcf865e51be19040c150560ceef6d3b668b63cb77b8b46e027c35b594cb9cbb03	0.00215BNB ($0.52)
Figure 1 uploaded as WebP file (resolution: 936 × 655; size: 13KB)	https://bscscan.com/tx/0xa8a8573a9be78ecc56a90a925a8015e222a0dc90b4531899ee3fc32bdef613c4	0.0009BNB ($0.22)
Figure 2 uploaded as WebP file (resolution: 665 × 400; size: 10.7KB)	https://bscscan.com/tx/0xf6307b9d834c40c87628446c4fb60c5f6a681e24d534ed7bdabb34e41cf7e350	0.0007BNB ($0.19)
**Scientific images** [23] **(**10.1371/journal.pbio.3001161.g003**)**		
Scientific image sourced from a research article, uploaded as jpg (figure 3A1, tissue scale; resolution: 795 × 221; size: 49KB)	https://bscscan.com/tx/0xdd4d10e73572200759a9ced8e8ef5f6d401092af8f67475c39fcf9d2f870360d	0.0055BNB ($2.51)
Scientific image sourced from a research article, uploaded as jpg (figure 3A2, cellular scale; resolution: 388 × 387; size: 17.67KB)	https://bscscan.com/tx/0x4b651846b43f7424010a569f47ba7812318c21c3fa98b65d30d28cce4307184d	0.002BNB ($0.93)
Scientific image sourced from a research article, uploaded as jpg (figure 3A3, subcellular scale; resolution: 387 × 387; size: 20.33KB)	https://bscscan.com/tx/0x37fc74bbc631109f2a114721687ce31f2771ac30b2b7e1c390da4a2981222a09	0.0023BNB ($1.06)
Scientific image sourced from a research article, uploaded as jpg (figure 3A, whole image; resolution: 791 × 683; size: 95KB)	https://bscscan.com/tx/0x6831da5deb68315e37d238699c960a95f16623250c0a49c3e61f0c4768bec4a7	0.01077BNB ($5.38)
**Audio**		
Audio file: sound effect (46KB, wav format)	https://bscscan.com/tx/0x7824740920767e8265f2a571004b5f569cc45bea49f27d526a9cdfc67415b126	0.0052BNB ($2.62)
**Video**		
Video file: salamander limb regeneration (94.9KB, mp4 format)	https://bscscan.com/tx/0x0070b6e0a7c73da987fdc0449bbbc3e6278a41972fe2f3ea79e3b216d34258e0	0.0107BNB ($5.37)
**Research article** [32] **(**10.1186/1472-6947-5-12**)**		
Article PDF file (209KB) split into 3 files; this is part 1 (1.zip.001, 70KB)	https://bscscan.com/tx/0x4d6569b3d4baef47e294edd59288bc8a49b6ed76da55cda149aa5709b8cee11e	0.0047BNB ($1.29)
Article PDF file (209KB) split into 3 files; this is part 2 (1.zip.002, 70KB)	https://bscscan.com/tx/0x00a22330461da0b5189dcc66c29560bf2d05442a3a65cf647b6ca4b792354154	0.0047BNB ($1.29)
Article PDF file (209KB) split into 3 files; this is part 3 (1.zip.003, 19KB)	https://bscscan.com/tx/0x641cc6a6f0ca378a5a93b7c5f88b785eda8855dd0e5bf22b07f1fc40d4118a60	0.0012BNB ($0.35)
**Research article** [33] **(**10.1186/1472-6947-7-24**)**		
Article PDF file (198KB) split into 2 files; this is part 1 (2.zip.001, 70KB)	https://bscscan.com/tx/0x56a2d81dc5ff4dc8b97e1d944559a567d89352cdc58f8120a8797bc67734c8cd	0.0047BNB ($1.42)
Article PDF file (198KB) split into 2 files; this is part 2 (2.zip.002, 63KB)	https://bscscan.com/tx/0x7406df6a5501d6737157f63ad73ac5e3d5d493980d6f9390832e3d68daf74acc	0.0042BNB ($1.27)
**Research article** [34] **(**https://doi.org/10.1016%2FS1473-3099(11)70092-5**)**		
Article PDF file (107KB) split into 2 files; this is part 1 (3.zip.001, 70KB)	https://bscscan.com/tx/0x40a8e5391ac082655ec4febf46b7914cf015034072940ee3d69a0ebda5f9849f	0.0047BNB ($1.42)
Article PDF file (107KB) split into 2 files; this is part 2 (3.zip.002, 31KB)	https://bscscan.com/tx/0x87b1d052fe6b7690b9398bfbd8773060170470c8da374e640f271d190e1e5564	0.002BNB ($0.62)
**Research article** [35] **(**https://doi.org/10.3390%2Fv5020753**)**		
Article PDF file (165KB) split into 2 files; this is part 1 (4.zip.001, 90KB)	https://bscscan.com/tx/0xf72b13325c912019641c97ceca8b13c1bc3633030ab7ade6cd4e92107f0be067	0.0061BNB ($1.82)
Article PDF file (165KB) split into 2 files; this is part 2 (4.zip.002, 62KB)	https://bscscan.com/tx/0x3b637d54246330c3e9127597f1dcc2fa7d1d0a720266e2a923bc6c8b8ecb18e9	0.0042BNB ($1.25)
**Research article** [24] **(**https://doi.org/10.7150%2Fjca.9971**)**		
Article PDF file (132KB) split into 2 files; this is part 1 (5.zip.001, 70KB)	https://bscscan.com/tx/0x8b12fb0662f26266bcf239455966f372a803bab2dce017222b61d28e8191a5f1	0.0047BNB ($1.42)
Article PDF file (132KB) split into 2 files; this is part 2 (5.zip.002, 55KB)	https://bscscan.com/tx/0xacc6cf7c9f6decf5d1002842424be9919709258a2a26428726e65675a7912875	0.0036BNB ($1.10)
**Encrypted document**		
Sample encrypted document of sensitive data	https://bscscan.com/tx/0x3ea60e3b37c8565481bedb57b36ab0f81bf5c58ae9008703975b4a5afd7fb038	0.00039BNB ($0.09)

Fees are indicated in U.S. dollars according to the BNB/USD exchange ratio at the time of transaction. Given the BNB and gas price fluctuations, the cost of storage can vary depending on the time of transaction. The average time for having a transaction confirmed on BNB Smart Chain was around 15 seconds. The TxID is included in the chain explorer link

### Overcoming the file size limitations for uploading files on-chain with INNBC DApp

**Fig. 10:**
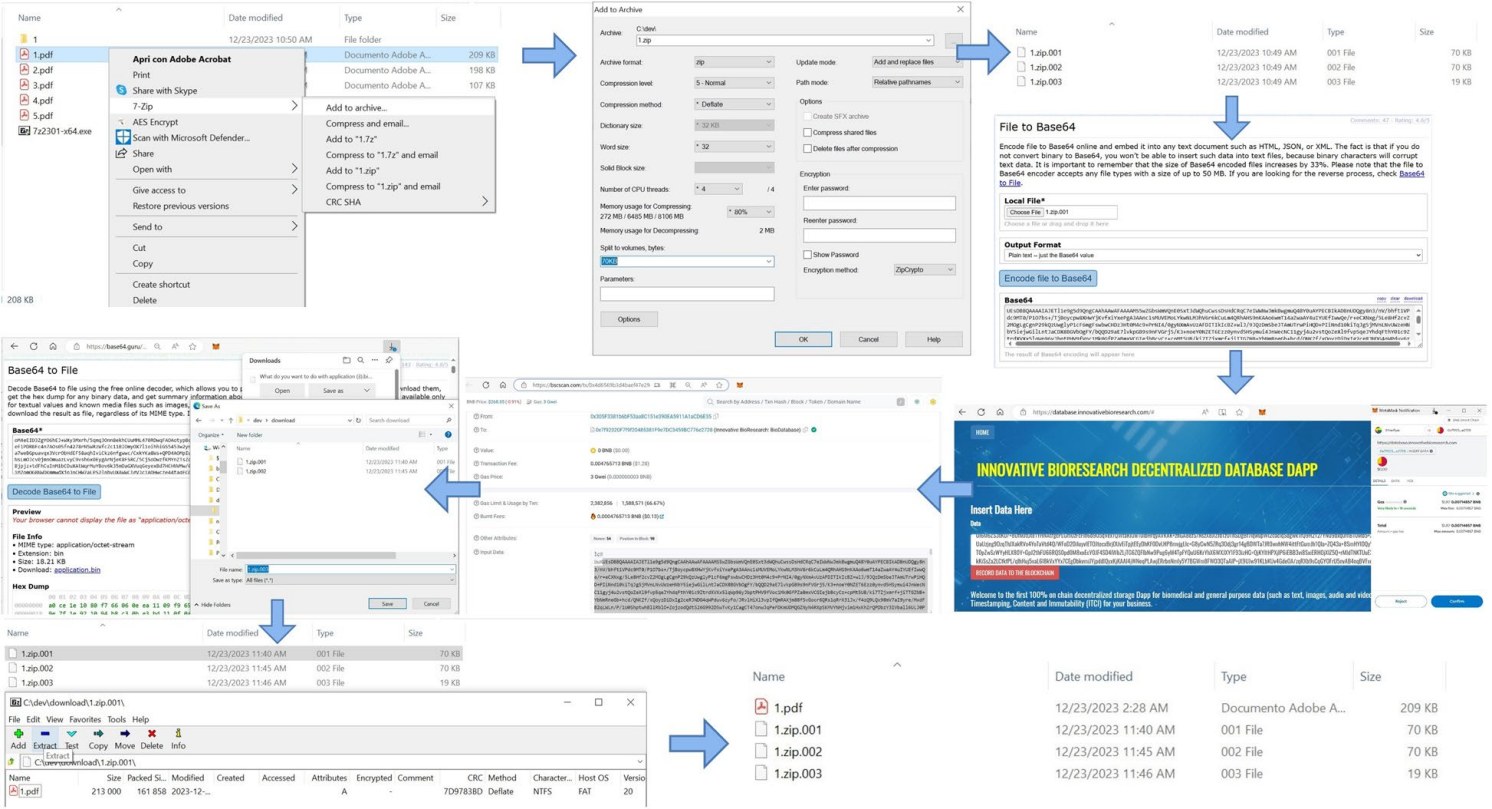
Overcoming the 95KB size limit to upload larger files on the blockchain. For this operation, we break up the file into chunks smaller than 95KB; they are posted on the blockchain as a multi-part archive file, which can be downloaded to recreate the original file. First, download “7-Zip” open-source file archiver (https://www.7-zip.org/). Right-click on the file, go to “7-Zip” and click on “Add to archive”, which will open the application interface and present the compression options: select “zip” as archive format (the most widely used format), leave the other settings as default, then go to “split to volumes, bytes:” where we can specify the split size, set a value less than 95KB (e.g., 70KB) and click on “OK”. The program will compress and break up the file into multiple parts, which will be saved in the same location as the original file. The files will be automatically named with an incrementing numbering system (e.g., “1.zip.001”, “1.zip.002”, “1.zip.003”), which is essential for successful merging of the files. We can now convert them to Base64-encoded text (using a free tool, e.g., https://base64.guru/) and use INNBC DApp (https://database.innovativebioresearch.com/) to upload the Base64-encoded files to the blockchain. Next, browse to the transaction page in the chain explorer and retrieve the data from the “input data” of the transaction. In order to copy the data correctly, we can exclude the initial external characters that are not part of the Base64-encoded data by pressing “Decode Input Data”, which will show us exactly where our data start. As shown in the figure, after we copy the data correctly, we can decode the data back from Base64 to file using the same free Base64 tool (https://base64.guru/) and download the files. Here, it is important to rename the files exactly with their original filenames (e.g., “1.zip.001”, “1.zip.002”, “1.zip.003”) and save them as “All files” with no extension, as shown in the figure. This is required for successfully merging the archive parts and extracting the original file. After downloading all the files and renaming them with the correct filename, we can right-click on the first file on the list according to their numbering system (e.g., “1.zip.001”), go to “7-Zip”, and click on “Open archive”, which will open the program interface, showing us a preview of the reconstructed file. We can then click on “Extract”, and the program will merge the archive parts, extracting the original file and saving it in the selected location

As previously mentioned, when uploading files to the blockchain using INNBC DApp, we experienced a file size limitation for each individual upload. We experienced “oversized data” error when attempting to upload files larger than 95KB that were posted on the blockchain as Base64-encoded data, resulting in the transaction not being executed (Fig. 7). Oversized data error occurs when the size of the data included in a transaction exceeds the upper limit allowed by the network. Considering that Ethereum blockchain currently has a limit of 128KB as the maximum size of input data allowed in a single transaction, it is very likely that BNB Smart Chain has a similar restriction. This is probably a preventive measure against spam and denial-of-service (DoS) attacks on the network. Given that Base64 encoding results in a 33% file size increase, a 95KB file upload would result in approximately 126KB (95KB plus 33%) of Base64 data, which is in accordance with a 128KB single transaction limit on BNB Smart Chain. We previously suggested that to overcome this limitation and upload files larger than 95KB, a possible solution is to split the file into smaller chunks and upload them separately to the blockchain. Such a task requires the possibility to recombine the data chunks back into the original file from the data posted on-chain. We wanted to evaluate the technical feasibility of this approach. Therefore, we decided to attempt to post on the blockchain several PDF files of research articles weighing more than 95KB [24, 32–35]. The articles were uploaded on-chain as multi-volume archives split into multiple files weighing less than 95KB. We used the free and open-source “7-Zip” application (https://www.7-zip.org/) as a file archiver tool. A file archiver is a computer program that splits a single file into multiple files as well as combining different files into a single file. They are stored in compressed containers known as “archives”. One of the advantages of using a file archiver is that, in addition to allowing the user to split a single file into smaller chunks, it also compresses the data, reducing the space required to store the file on-chain, which can save on gas fees. The compression is accomplished by searching for similarities in the data to rewrite the file in a more efficient format by removing redundant data. This is a lossless compression, meaning that it is possible to reverse the process and reconstruct the original information by decompressing the archive. As such, this is an ideal process for biomedical data to avoid losing information. Starting with the largest PDF file on the list [32], as a first step, we split it up into smaller chunks using the 7-Zip tool. We used the “zip” archive format because it is the most common and easily accessible format for most users. The size of the PDF file is 209KB, and we selected to split it into 70KB volumes, which created three files, two with the same size (70KB) and one corresponding to the remaining size (19KB). We ended up with three files, named “1.zip.001”, “1.zip.002”, and “1.zip.003”, from the original “1.pdf” file. This will be crucial later on since we need to carefully rename the three files to their original filenames after downloading them from the blockchain in order to successfully reconstruct the original PDF file. We then proceeded to encode the three part files to Base64 and posted them on the blockchain with INNBC DApp: 1.zip.001 (size: 70KB, TxID: https://bscscan.com/tx/0x4d6569b3d4baef47e294edd59288bc8a49b6ed76da55cda149aa5709b8cee11e, cost: 0.0047BNB/1.29USD), 1.zip.002 (size: 70KB, TxID: https://bscscan.com/tx/0x00a22330461da0b5189dcc66c29560bf2d05442a3a65cf647b6ca4b792354154, cost: 0.0047BNB/1.29USD), 1.zip.003 (size: 19KB, TxID: https://bscscan.com/tx/0x641cc6a6f0ca378a5a93b7c5f88b785eda8855dd0e5bf22b07f1fc40d4118a60, cost: 0.0012BNB/0.35USD). The next step involved downloading the files from the blockchain and joining them to extract the original PDF file. As previously mentioned, to perform this operation, we need to search for the transaction on the chain explorer, where we can view the transaction input data and convert it into readable text (by selecting “view input as, UTF-8”). Additionally, we can also see exactly where our data start in order to copy only the Base64-encoded text belonging to our file (by pressing “decode input data”). At this point, we converted the Base64-encoded data back to file and, as previously mentioned, it is crucial that the generated files are saved with their exact original filenames for the joining process to be successful. In addition, files must be saved as “All files” with no extension. Next step is to open the first file on the list with the archiver tool (right-click on the file, go to “7-Zip” and click on “Open archive”), which shows us a preview of the reassembled file with its full name and size; then, we execute the “extract” function of the program and point to the folder where we want the file to be extracted. This resulted in the successful reconstruction of the original “1.pdf” 209KB file (all steps are illustrated in Fig. 10). We then successfully repeated the same procedure for the other PDF article files [24, 33–35] (all data reported in Table 1). This demonstrates that one of the primary drawbacks of the proposed tool can be overcome by using this method to store files larger than 95KB on-chain. Therefore, we demonstrate that storing on-chain files of arbitrary size with INNBC DApp is technically possible, although the main limitation will be the cost of the gas fees. The decentralized storage solution offered by INNBC DApp, however, is meant to be used with relatively small biomedical data files, such as documents and images, that are typically found in scientific publications. It is not meant to replace traditional centralized data storage for storing very large files.

### Dealing with sensitive data on the blockchain

**Fig. 11:**
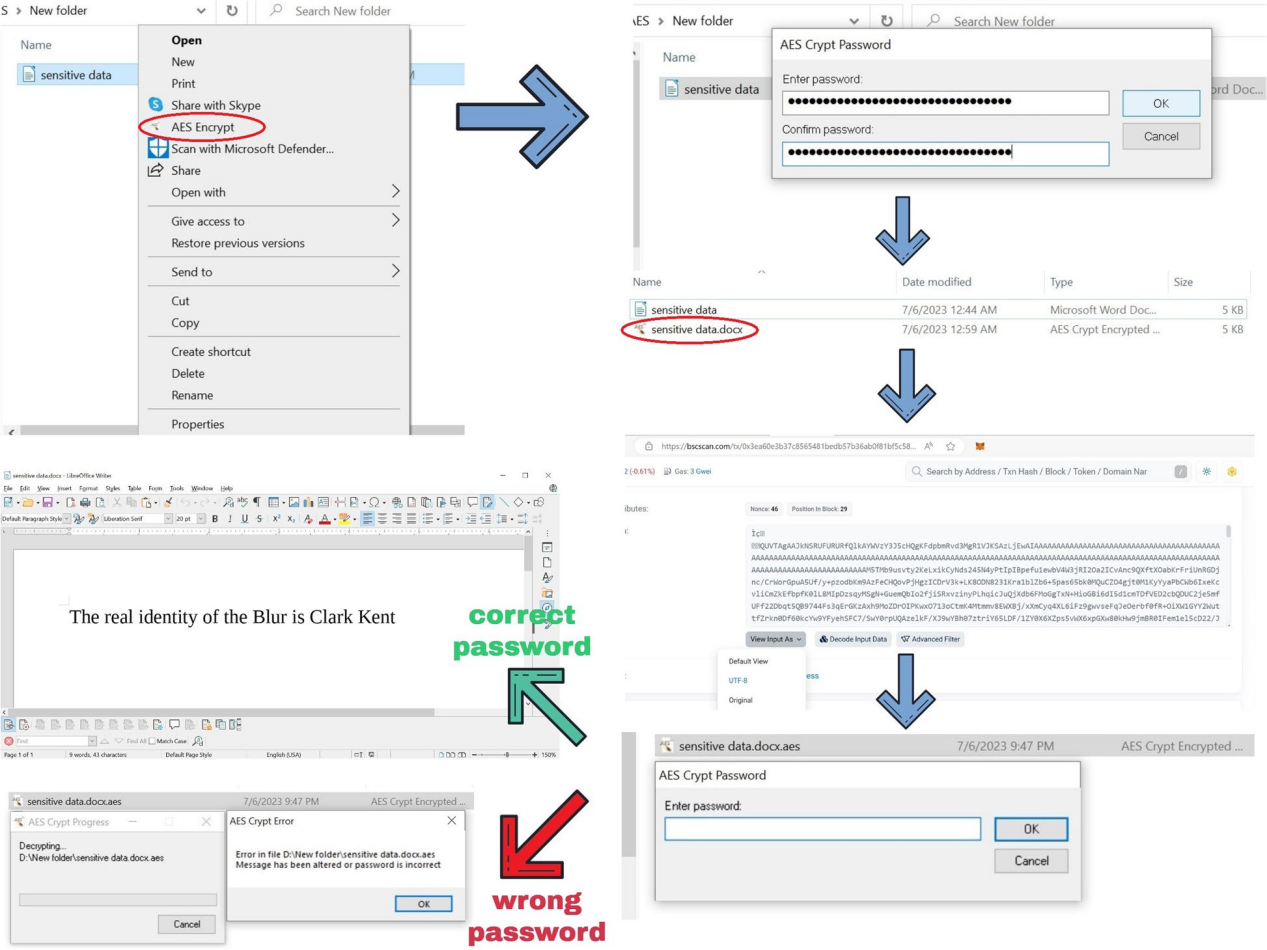
Posting AES-encrypted documents on the blockchain to protect the privacy of sensitive information. First, the document is encrypted with a highly secure password (AO6kvkm6iR9nWFBjLuYf1HyJvEXslIGo). The encrypted document is then converted to Base64 and posted on the blockchain (https://bscscan.com/tx/0x3ea60e3b37c8565481bedb57b36ab0f81bf5c58ae9008703975b4a5afd7fb038). The Base64-encoded file can then be sourced from the transaction data and converted back to the original encrypted file. After recreating the encrypted file, it is only possible to decrypt the file and generate the original document with the correct password. It is important to rename the file to its correct extension after decoding Base64. For instance, we renamed the encrypted document file to the “.aes” extension, and after decrypting the file, it was renamed to the original “.docx” extension. For this procedure, we used AES Crypt for encrypting the document (https://www.aescrypt.com/), Base64 guru for Base64 encoding and decoding (https://base64.guru/), and INNBC DApp for posting the data on the blockchain (https://database.innovativebioresearch.com/). All of these are freely available tools

In the previous scenario, we uploaded data from scholarly journals and other sources that are intended to be accessible to the public. This represents an ideal application for posting data on a public blockchain. But there are also specific applications that require special attention, including when handling sensitive privacy information that can be found in healthcare data. Here, we wanted to address the issue of how to post on-chain sensitive data while at the same time ensuring privacy protection and compliance with regulations, such as GDPR, with a real-world example transaction using INNBC DApp. In fact, we believe that it is possible to take advantage of the decentralization and security of a public blockchain in this scenario by using data encryption as a privacy-preserving processing technique for complete data anonymization. Data encryption is a process that uses an encryption key and an algorithm to encode the original readable data into an unreadable format called ciphertext. Only the encryption key, which the person encrypting the data owns, can decode ciphertext back to its original format. This ensures that without the key, it is not possible to decipher a ciphertext and access the original information. The AES encryption algorithm has been proposed to secure medical health records in hospital systems that use cloud storage and need to protect sensitive data [36]. Therefore, we decided to use AES Crypt (https://www.aescrypt.com/), an open-source AES file encryption software using a powerful 256-bit encryption algorithm, in combination with INNBC DApp. We created a sample document file containing the text “The real identity of the Blur is Clark Kent” that was encrypted using a highly secure password (AO6kvkm6iR9nWFBjLuYf1HyJvEXslIGo), encoded to Base64, and posted on the blockchain with INNBC DApp (https://bscscan.com/tx/0x3ea60e3b37c8565481bedb57b36ab0f81bf5c58ae9008703975b4a5afd7fb038). The encrypted document can be reconstructed by decoding the Base64-encoded text stored in the transaction. As previously mentioned, it is important to rename the file to its correct extension after decoding it. In this case, we renamed the encrypted document file to the “.aes” extension, and after decrypting the file, it was renamed to the original “.docx” extension. All steps are illustrated in Fig. 11. Here, we demonstrate that we shared a sample document of sensitive data that is totally inaccessible without access to the password. As such, this is a real-world example of how to post data on a blockchain with an effective privacy protection solution. We believe this is a suitable solution for the protection of data privacy. As previously mentioned, AES encryption is already used to protect cloud-stored healthcare records in hospital systems [36]. The advantage of using a blockchain with respect to cloud-based storage is that the data can never be modified or tampered with in any way.

## Conclusions

Here, we document the first DApp designed for storing biomedical data on a public blockchain, performing actual transactions, and analyzing the associated network gas fee costs. We therefore provide results in terms of transaction data generated on a public distributed ledger such as BNB Smart Chain, with such data permanently recorded and publicly available to the scientific community for further analysis. Unfortunately, to the best of our knowledge, no previous relevant work in the field has produced actual transaction data that we can directly compare with our results [16–18]. However, some comparisons and considerations can be made in terms of software design with existing blockchain tools and methods proposed for managing biomedical and health data. Johnson et al. [19] describe how to possibly build a decentralized application for sharing geolocation data between study participants and research teams. Unfortunately, they present no live, working version of the DApp and just provide the source code that only an experienced developer can piece together to produce an actual working DApp. There is no real-world testing of the DApp in the study, no actual deployment of smart contracts on a public mainnet blockchain, and no actual transaction data to analyze. Such data can be very important, especially for analyzing the cost in terms of gas fees and indicating whether it is economically feasible to use a proposed DApp for the intended use case. Comparing the UI, the front-end is presented as an iOS iPhone application, which is limited to the input of geolocation data. By contrast, our DApp allows the input of different data formats (e.g., documents, images, audio, and video) and their decentralized storage in addition to not being restricted to running on a closed, proprietary mobile phone operating system, as we developed a web-based DApp requiring only a web browser with an integrated Web3 wallet. We believe our design choice provides a more open, accessible, permissionless, and trustless solution, taking full advantage of Web3 technology. Another issue is that the proposed iOS application manages to store user wallet addresses and the associated private keys directly, meaning that users have to input such sensitive data directly into the application. This poses a major security risk as the application could be compromised and such data stolen through hacking, meaning that hackers could then access user wallet funds and steal any valuable currency held there. By contrast, our DApp never stores or requires users to enter their private keys, as users use a decentralized Web3 wallet (e.g., Metamask) as an access point for DApp interaction. For maximum security, users can also use a cold hardware wallet to log into their Web3 wallets, storing private keys offline and ensuring that no malicious actor on the internet can access them. Other blockchain-based tools, such as MedRec, Ancile, and OmniPHR [16], are specifically aimed at managing healthcare records and use smart contracts and blockchain for managing permission to access the data and proof-of-validity rather than for storing the data themselves. In such cases, the only data stored on-chain are blockchain anchors, while the actual healthcare records are stored off-chain. As such, we must rely on storage solutions external to the blockchain for the availability of the documents without taking advantage of its immutability for the raw data. By contrast, with INNBC DApp, we take full advantage of immutability by storing all data on-chain, with a true decentralized storage solution. It should be noted that we are proposing this application to store data that are normally shared in scientific publications and are therefore meant to be public. In fact, we stored data from previously published scientific articles. Special attention is needed with healthcare data containing sensitive information. To protect patient privacy, if this application is used for such data, it must be used in compliance with current regulations, such as the GDPR. Here, we provide a real-world example of how the privacy issue could be solved by encrypting the data with an encryption algorithm, making the information unreadable to the public without the encryption key. Accordingly, it must also be mentioned that we only provide an instrument, and it is the user’s sole responsibility to use the DApp according to the existing regulations and laws. A summary of the advantages of INNBC DApp for decentralizing biomedical data storage and sharing with respect to the existing methods and tools is presented in Table 2.

**Table 2:** Comparing the advantages of INNBC DApp with respect to existing tools for decentralizing data storage and sharing

Proposed Tool	Web3-based Permissionless DApp	Decentralized Data Storage	Decentralized Data Sharing	Available Transaction Data	Scalability
INNBC DApp	Yes	Yes. Text, images, audio, video, and any Base64-encoded file	Yes	Yes, on BNB Smart Chain blockchain	95KB max per single upload, 0.06–0.1BNB gas fee per MB
Johnson et al. [19] (DApp)	No	No	No	No	NA
Other blockchain-based tools for health data (e.g., MedRec, Ancile, OmniPHR [16])	No	No, raw data are stored off-chain (only blockchain anchor stored on-chain)	No	No	NA

Web3-based permissionless DApp: featuring a decentralized Web3 interface not requiring trust by the user to access the DApp. Decentralized Data Storage: different data formats (e.g., documents, images, audio, video) are fully stored on a public blockchain, ensuring permanent availability. Decentralized Data Sharing: raw data are fully stored and freely available on a public blockchain. Available Transaction Data: actual transaction data have been generated by using the DApp and are permanently available on a public blockchain for further analysis by the scientific community. Scalability: maximum size of allowed data stored on-chain with a single transaction (95KB of original data size with Base64 for INNBC DApp) and storage cost per megabyte (0.06–0.1BNB/14–23USD per MB for INNBC DApp). BNB: Binance Coin. MB: megabyte. KB: kilobyte. NA: not available

In this study, we assume that it is possible to store actual biomedical data fully on-chain and explore this approach. We start by posting on the blockchain a previously published research study, using the strategy of uploading the data directly as raw text and numerical data. When figure data are represented only by graphs, uploading the original raw numerical data instead of the corresponding image files is a possible strategy to save on storage space. In such cases, we may have originally had very large, high-resolution images to allow readability of the graphs, as in our case, and using the raw numerical data instead is a solution to save on gas fees. Next, we tested whether it is possible to upload actual image files on-chain and the possible limitations. Accordingly, we used scientific images sourced from a paper discussing how to properly create images suitable for scientific articles [23]. First, we extracted the three singular photographs from the article’s figure 3A into three different jpg files weighing 49KB, 17.67KB, and 20.33KB and posted them on the BNB Smart Chain blockchain. These small sizes were enough to maintain the original image quality and clarity, even with the additional image compression we used. We then put the whole article’s figure 3A into a single image file weighing 95KB as the largest size allowed for a single upload, showing that even within these limitations, it is still possible to include enough image data to be useful for the purpose of storing and sharing scientific images on a blockchain. If we need to upload files larger than 95KB, we can split the data into smaller chunks and upload them as a multi-part archive. We successfully demonstrated the technical feasibility of this approach. This means that, theoretically, there is no hard cap on the amount of data that can be stored on-chain; however, in terms of scalability, the main factor limiting decentralized storage is gas fee cost, which was calculated to be 0.1BNB per megabyte of data (23USD per MB). Although in some instances the cost was even lower, 0.06BNB per megabyte (14USD per MB), due to the fluctuations in gas and BNB coin prices. Realistically, this means that we can successfully use this application to store relatively small chunks of data, such as those included in most scientific articles, at a reasonable cost. To demonstrate this, we successfully posted several complete research articles on the blockchain. A blockchain is not intended to replace traditional storage solutions but rather to create a permanent record of relatively small chunks of data organized as a chain of data blocks. This can be an ideal application for the data usually found in scientific articles, as we demonstrate here. The average transaction confirmation time was approximately 15 seconds, and retrieving data is almost instantaneous from the transaction page on the block explorer, supporting the conclusion that INNBC DApp is a real-world usable tool. Additionally, we tested the possibility of storing audio and video files on-chain. Although we may face greater limitations here due to the generally larger size of such files, which can result in expensive gas fees, we show that it surely is possible to store them on-chain. Using Base64 in combination with INNBC DApp opens the door for storing a very large number of different file formats, which is very exciting. In fact, we had no issue posting on the blockchain AES-encrypted files to protect sensitive data. Once posted on-chain, data have a unique transaction hash (TxID) and a timestamp, providing proof of authorship for those authors looking to protect their work from being stolen or manipulated. A blockchain is censorship-resistant, meaning that once stored on-chain, data are permanently available and cannot be deleted, altered, or obscured from the public. A blockchain is public, meaning that there are no restrictions on accessing the data. In a blockchain, there is only a one time fee to pay for storing the data, after which it is the chain itself that will ensure permanent data storage, and no further cost or subscription is needed. Reading the data is also free on a blockchain. With all these considerations in mind, we conclude that blockchain technology has a real use case for creating permanent records of biomedical data and sharing them, especially when using a modern, proof-of-stake blockchain such as BNB Smart Chain. In terms of scalability, our solution relies on the capabilities of the BNB Smart Chain blockchain, which features impressive performance with an average block time of approximately 3 seconds and a transactions per second (TPS) output up to 188TPS, measured in real-world network usage according to the chain explorer data (Fig. 1). We can assume these numbers are far from full network usage. Considering that the chain can handle high volumes of transactions with no network congestion, especially with intensive DeFi DApps by many crypto projects, we can also assume that the impact of the scientific community uploading biomedical data would be completely sustainable. By contrast, in its current state, the Ethereum network is unable to serve the same use case at a reasonable gas fee cost (Fig. 1), and we necessarily need to wait for upgrades such as sharding to be implemented before transaction fees can be drastically reduced. Sharding involves splitting the Ethereum network into multiple parallel subchains called “shards” [8]. Scaling the main chain, such as with the planned Ethereum sharding update, is defined as “layer-1” scaling. Building on top of the main chain is defined as “layer-2” scaling. Layer-2 solutions are high-performance protocols that perform off-chain transactions and then broadcast the final result to the layer-1 chain. Examples of layer-2 solutions include “state channels” and “rollups” [8, 37]. Layer-2 scaling can exponentially increase the scalability of a blockchain, but at the expense of decentralization. In this regard, we believe that layer-1 is more suitable for our DApp because it offers the greatest decentralization and data immutability, whereas layer-2 provides the benefit of extremely low gas costs but at the expense of decentralization. Given that we are proposing to store data on-chain, such as documents and images, we also need to consider the possibility that malevolent actors could flood the network with random data. Actually, layer-2's incredibly low fees might be a double-edged sword because they would make spamming attacks much less expensive as well, making the chain much more vulnerable to an influx of “garbage” data. By contrast, layer-1 would require significantly higher fees to generate the same amount of spam transactions, effectively discouraging such attacks. In order to allow for reasonable storage costs, transaction fees must be low enough, but not so low as to encourage malicious traffic attacks. For this reason, we believe the BNB Smart Chain layer-1 network is currently the best option for INNBC DApp. In conclusion, we propose INNBC DApp as a readily available, decentralized software tool for blockchain-based biomedical data storage, aiming at increasing user adoption of Web3 and blockchain technology by the scientific community. This is one of the important goals of INNBC project that we aim to further expand in the future with the development of INNBC Smart Chain, our own blockchain network that will be devoted to decentralized science [20].

## Data Availability

Project name: INNBC DApp. Project home page: https://www.innovativebioresearch.com/. DApp direct link: https://database.innovativebioresearch.com/. Operating system(s): platform independent. Programming languages: JavaScript, HTML, Solidity. Other requirements: Web/DApp browser with a Web3 wallet (e.g., Metamask). License: GNU GPLv3. Data sharing statement: the relevant data for this work are within the paper. The transaction data generated by this study are permanently stored and publicly available on the BNB Smart Chain blockchain with the TxIDs and links provided in the paper.
